# Allo-priming as a universal anti-viral vaccine: protecting elderly from current COVID-19 and any future unknown viral outbreak

**DOI:** 10.1186/s12967-020-02363-3

**Published:** 2020-05-12

**Authors:** Michael Har-Noy, Reuven Or

**Affiliations:** 1 Cancer Immunotherapy and Immunobiology Center, Hadassah-Hebrew University Medical Center, 9112001 Jerusalem, Israel; 2 Immunovative Therapies, Ltd, Malcha Technology Park, B1/F1, 9695101 Jerusalem, Israel; 3 Mirror Biologics, Inc., 4824 E Baseline Rd #113, Phoenix, AZ USA

**Keywords:** COVID-19, Immunosenescence, Inflammaging, Cell therapy, Immunotherapy, Vaccine

## Abstract

**Background:**

We present the rationale for a novel allo-priming approach to serve the elderly as a universal anti-virus vaccine, as well serving to remodel the aging immune system in order to reverse immunosenescence and inflammaging. This approach has the potential to protect the most vulnerable from disease and provide society an incalculable economic benefit. Allo-priming healthy elderly adults is proposed to provide universal protection from progression of any type of viral infection, including protection against progression of the current outbreak of COVID-19 infection, and any future variants of the causative SARS-CoV-2 virus or the next ‘Disease X’. Allo-priming is an alternative approach for the COVID-19 pandemic that provides a back-up in case vaccination strategies to elicit neutralizing antibody protection fails or fails to protect the vulnerable elderly population. The allo-priming is performed using activated, intentionally mismatched, ex vivo differentiated and expanded living Th1-like cells (AlloStim^®^) derived from healthy donors currently in clinical use as an experimental cancer vaccine. Multiple intradermal injections of AlloStim^®^ creates a dominate titer of allo-specific Th1/CTL memory cells in circulation, replacing the dominance of exhausted memory cells of the aged immune system. Upon viral encounter, by-stander activation of the allo-specific memory cells causes an immediate release of IFN-ϒ, leading to development of an “anti-viral state”, by-stander activation of innate cellular effector cells and activation of cross-reactive allo-specific CTL. In this manner, the non-specific activation of allo-specific Th1/CTL initiates a cascade of spatial and temporal immune events which act to limit the early viral titer. The release of endogenous heat shock proteins (HSP) and DAMP from lysed viral-infected cells, in the context of IFN-ϒ, creates of conditions for in situ vaccination leading to viral-specific Th1/CTL immunity. These viral-specific Th1/CTL provide sterilizing immunity and memory for protection from disease recurrence, while increasing the pool of Th1/CTL in circulation capable of responding to the next viral encounter.

**Conclusion:**

Allo-priming has potential to provide universal protection from viral disease and is a strategy to reverse immunosenescence and counter-regulate chronic inflammation (inflammaging). Allo-priming can be used as an adjuvant for anti-viral vaccines and as a counter-measure for unknown biological threats and bio-economic terrorism.

## Background

Herein we propose to use a novel allo-priming strategy using patented, allogeneic Th1-like immune cells conjugated to CD3/CD28 microbeads (AlloStim^®^) to serve as a “Universal Anti-Viral Vaccine” to protect the health of elderly adults. Vaccination has been a strategy to protect against viral diseases in adults, such as influenza, pneumococcal pneumonia, shingles and hepatitis A/B. Successful prophylactic vaccination mechanisms provide protection through eliciting neutralizing antibodies to prevent viral entry into cells. However, this strategy does not provide protection against antigenic shift or drift variants of the original virus [1, 2]. Currently, there are at least three known variants of the SARS-CoV-2 virus [3].

In addition, pathological viruses are intracellular and not always accessible to antibodies. For this reason, neutralizing antibody vaccines have not been effective against a number of complex viruses, including HIV, HCV, CMV, Zika, RSV, Dengue and SARS/MERS. For the same reason, convalescent serum prophylaxis and treatment may not be able to confer sterilizing immunity or memory. These sophisticated viruses may require an effective cellular immune response for sterilizing immunity [4–8]. Without sterilizing cellular immunity, there can be viral recurrence as has been reported with COVID-19 [9].

Many efforts are underway to develop anti-viral vaccines which elicit protective cellular immunity [10], but these have not yet been successfully translated to demonstrate clinical benefit [11]. The age-related functional decline in cellular immunity (immunosenescence) makes the elderly less able to mount a cellular immune response to vaccination, making this population more vulnerable to morbidity and mortality associated with viral diseases and less likely to respond to an anti-viral vaccine.

In addition, elderly also suffer detrimental effects on their immune function due to chronic inflammation, known as “inflammaging” [12]. Inflammaging is correlated with comorbidities such as cancer, arthrosclerosis, neurodegenerative diseases (e.g., Alzheimer’s and Parkinson’s disease) all which increase the likelihood of serious progression of viral infection. In addition, the aging of the structure and function of the lungs contributes to increased incidence of pneumonia, acute respiratory distress syndrome (ARDS) and sepsis in the elderly after respiratory viral infection.

The remodeling of the senescent immune systems of the elderly through allo-priming is proposed as a method to restore cellular immune function in this population. The ability to restore functional cellular immunity to the elderly can increase responsiveness to viral infections, including COVID-19 and any future emergent novel virus. In essence, an elderly immune system modulated by allo-priming would potentially respond to viral infection in a similar manner to the immune response of younger individuals, resulting in less serious disease. The immunomodulation of the elderly immune system to function more like a youthful immune system should also restore responsiveness to any current or future viral-specific vaccines. A more balanced immune system in the elderly can also counter-regulate inflammaging, providing broad ranging health benefits to the elderly and to society [13].

## Main text

### Allopriming

The allo-priming concept is designed to prime the immune systems of healthy elderly adults to create high titers of allo-specific Th1/CTL memory cells which can become activated upon encounter with any virus (by-stander activation) [14]. The by-stander activation of allo-specific Th1/CTL memory cells upon viral encounter causes the release of interferon-gamma (IFN-ϒ), which creates an “anti-viral state” [15]. IFNs create an antiviral state in both virus-infected cells and uninfected, bystander cells, by inducing a program of gene transcription that interferes with multiple stages of viral replication cycles through various mechanisms [16].

The IFN-ϒ release from by-stander activated allo-specific Th1/CTL memory cells activates innate effector cells (e.g., NK, NKT and macrophages) which in turn release additional IFN-ϒ, sustaining the anti-viral state. These activated innate effector cells lyse viral infected cells, controlling acute viral burden. By-stander activation of resident allo-specific CTL can also cross-react to lyse viral infected cells [17]. The lysis of viral infected cells by activated innate effector cells and cross-reactive allo-specific memory CTL releases “danger signals” [18] and heat shock proteins (HSP) [19] which chaperone viral antigens (e.g., GRP78, HSP70) [20, 21] into the microenvironment, creating the conditions for “in situ vaccination”, which leads to development of viral-specific cellular immunity.

Antigen presenting cells (APC), such as dendritic cells (DC), engulf and process released HSP-chaperoned viral antigens. Processing in the context of danger signals and IFN-ϒ, causes the maturation of immature DC to IL-12+ DC [22, 23]. IL-12 production acts to further increases IFN-ϒ, sustaining the anti-viral state and upregulating MHCI, MHCII and co-stimulatory molecules on APC [24, 25]. The mature DC migrate to regional lymph nodes and display viral antigens on MHC I and MHC II with co-stimulator CD80/86 expression, leading to viral-specific Th1/CTL expansion. The IFN-ϒ upregulates MHCI on viral infected cells so these cells can be recognized and killed by viral-specific CTL [26]. These viral-specific CTL can then orchestrate a sterilizing immune response and later differentiate into memory cells that provides protection from re-infection.

Thus, allo-antigenic priming can modulate the systemic immune balance and provide a pool of non-exhausted allo-specific Th1/CTL memory cells capable of rapidly responding to viral infection. The non-specific activation of these allo-specific memory cells upon viral encounter causes release of IFN-ϒ. The release of IFN-ϒ orchestrates the sequential activation of immune cells resulting in formation of an antiviral state, innate elimination of invading viruses and development of a viral-specific effector response and memory.

Each time this cascade is initiated by viral encounter, the virus elimination serves as a booster to the original allo-antigenic priming. The new viral-specific Th1/CTL memory cells resulting from viral elimination join the resident memory allo-specific Th1/CTL resulting from the allo-antigenic priming, increasing the titer of Th1/CTL memory cells in circulation. Encounter with each new virus will non-specifically activate all previous non-exhausted memory cells and support the immune cascade that eliminates the new virus. Memory Th1/CTL to each new virus, in turn, increases the titer of Th1/CTL memory cells primed to respond to a subsequent viral encounter.

Creating a memory Th1/CTL immune response to alloantigen thus provides protection against an unrelated viral infection through the immune mechanism known as “heterologous immunity”. Heterologous immunity occurs when by-stander activation of immune memory cells to one virus alters the immune response to, and the course of infection of, an unrelated virus encountered later [27, 28].

In this manner, healthy adults can be provided pan-viral protection against viral infections, including SARS-CoV-2, influenza A/B and any future variants and unknown novel viruses that may emerge. This self-amplifying process can provide long-term disease mitigation and universal protection against known and unknown viral infections for the elderly.

### Immunity to alloantigens

In order to effectuate the anti-viral and anti-aging immune mechanisms, the allo-priming must elicit allo-specific Th1/CTL immunity. Injection of alloantigen alone can elicit humoral and Th2 responses, and multiple injections can result in tolerance to the allo-antigens. Many factors, both intrinsic and extrinsic to the allograft, can influence the nature and magnitude of the allo-rejection immune response, including the nature of the allograft, the site of the body where it is placed, and the immunological status of the recipient [29].

To assure consistent differentiation of allo-specific Th1/CTL memory after allo-antigen priming, regardless of host immune status and age, a bioengineered, patented, immune cell called “AlloStim^®”^ is used. AlloStim^®^ is living, activated, intentionally mismatched, ex vivo expanded and differentiated allogeneic Th1-like cells derived from healthy donors. Injection of undifferentiated allogeneic immune cells or non-activated allogeneic immune cells were not able to elicit allo-specific Th1 memory, while priming with activated allogeneic Th1-like AlloStim^®^ cells elicited dominant Th1 immunity and had both a protective effect and a therapeutic effect in a cancer model [30, 31].

AlloStim^®^ cells are administered intradermally (ID) in an activated state. To assure the injected cells are activated when administered, they are prepared with anti-CD3/anti-CD28-coated microbeads attached. The activated AlloStim^®^ cells are formulated as a frozen dosage form at 1 × 10^7^ cells/ml in PlasmaLyte A containing 1% human serum albumin and 2% DSMO. Individual doses of 0.5 ml are thawed and administered intradermally every 3–4 days for up to five total doses. This priming schedule is sufficient to provide high titers of circulating allo-specific Th1/CTL. AlloStim^®^ has been evaluated in Phase I/II, Phase II and Phase IIA clinical trials in USA and Thailand in chemotherapy-refractory metastatic solid tumor patients. Phase IIB pre-registration and Phase IIB/III randomized, controlled registration clinical trials are currently being prepared for launch in third line metastatic colorectal cancer and advanced/metastatic hepatocellular carcinoma in USA and Asia, respectively. In these human clinical trials, AlloStim^®^ has demonstrated ability to down-regulate checkpoint molecules in the tumor microenvironment, cause system-wide infiltration of effector T-cells and NK cells into metastatic lesions (conversion from “cold” to “hot”) and has consistently demonstrated a tail of between 20 and 35% of long-term survivors in chemotherapy-refractory metastatic disease with a good safety profile [32, 33].

Activated AlloStim^®^ express high density CD40L and type 1 cytokines, including IFN-ϒ, TNF-α and GM-CSF. AlloStim^®^ has been shown to modulate the immune systems of heavily pre-treated metastatic cancer patients (which resemble the senescent immune systems of the elderly) and has been used in protocols which were designed to elicit the same anti-tumor mechanism of allogeneic stem cell transplant procedures (graft vs tumor or “GVT”) without the need for a matched donor, chemotherapy conditioning or risk of GVHD [30, 31, 34–38].

Allogenic cells are highly immunogenic and are rejected even by immunocompromised hosts [39]. The rejection of the mis-matched cells eliminates risk of GVHD side-effects of allogeneic cell administration. Multiple ID injections of AlloStim^®^ amplify the titer of allo-specific Th1/CTL cells in circulation and a portion of these cells differentiate into long lasting memory cells. These allo-specific Th1/CTL memory cells provide a functional pool of immune cells that are capable of non-specific (by-stander) activation [40] upon encounter with virus [14, 41] resulting in immediate release of IFN-ϒ.

The production of proinflammatory cytokines by AlloStim^®^, including IFN-γ and TNF-α, initially activates host NK cells to reject the allogeneic cells, due to missing self-MHC I on the CD4+ AlloStim^®^ cells. The NK cell compartment is highly stable in terms of function and phenotype in the elderly [42] and can therefore readily reject mismatched allogeneic cells. In subsequent ID AlloStim^®^ injections, the production of IFN-ϒ and expression of CD40L by AlloStim^®^ activates macrophages that reject the AlloStim^®^ [43]. The rejection of the allogeneic cells by NK cells or macrophages in the skin results in release of endogenous HSP and DAMP danger signals [22], which in the context of CD40L and IFN-ϒ expressed by AlloStim^®^ result in DC maturation to IL-12+ DC1 phenotype and their migration to draining lymph nodes to interact with cognate T-cells to elicit allo-specific Th1/CTL immunity [44].

### Elderly adults

The current COVID-19 viral pandemic has led to high number of deaths worldwide. This is the third serious coronavirus (CoV) outbreak in less than 20 years, following SARS in 2002–2003 and MERS in 2012. Elderly people account disproportionately to the morbidity and mortality associated with CoV infection. Vaccines have been the main global strategy to minimize the impact of viral infections, but no successful vaccines have yet been developed for these previous highly virulent CoVs. In any event, elderly adults generally respond poorly to anti-viral vaccines [45–47]. This creates a high unmet medical need for a vaccine to provide protection to the elderly from the current COVID-19 pandemic and protect from any future pandemics.

While healthy younger adults generally present with more mild symptoms in response to viral infection, the elderly are slow to respond and are susceptible to higher viral titers and chronic viral infection which leads to progression to severe symptoms, especially in the setting of respiratory viral infections [48–54]. The delayed and poorly effective responsiveness of elderly to viral infection is due, in large part, to compromised cellular immunity [55–57] related to “immunosenescence” [58–60].

Elderly individuals have a significantly delayed innate immune response to viral infection and the adaptive immune response that follows is often sub-optimal [61]. The delayed innate immune response results in accumulation of high viral titers which, in turn, serves to elicit a late over-active immune response which is correlated with a more severe and often lethal clinical course [62]. The acute high viral levels that are achieved in elderly as a result of late innate immune response also increases the virulence of the virus, by increasing chances of human-to-human spread of infection.

There is a dysregulation in the Th1/Th2-system in the elderly, which is dominated by Th2-functions [63, 64]. Memory Th1/CTL cells in elderly adults are diminished and functionally exhausted [65–68]. Memory cells of the elderly become exhausted due to lifelong antigenic challenge which can be as a result of chronic viral infection (e.g., CMV, EBV, HBV, HCV) [69, 70]. Exhausted memory cells are unable to be non-specifically activated upon viral encounter. This defect in cellular immunity in the elderly limits the availability of heterologous immune mechanisms to react to acute viral infection, which contributes to a slower and often inadequate innate response to viral infection.

Elderly also have limited T-cell repertoires [71, 72] which inhibits their ability to develop viral-specific adaptive T-cell immune responses, both prophylactically (by vaccination) [73], and in situ (as a result of adaptive immune clearance of infection). This suppressed cellular immunity together with the aging of the respiratory organs makes elderly more susceptible to pathological effects of viral infection, especially effects of respiratory viral infection [74]. The immune defects of the elderly make this population less likely to benefit from vaccinations as preventative measures against infectious diseases [75].

The current inability to provide elderly with protection from viral infection has an enormous economic impact on society [76, 77]. The medical and economic impact of pandemics which disproportionately affect the elderly will be exacerbated as the proportion of the adult population increases globally. The current projections indicate that by 2050, the group of 60 years and older will represent 21.1% of the population [78]. Therefore, there is an urgent unmet need to develop strategies to reverse immunosenescence and to develop methods which provide protection of the elderly population from viral epidemics.

### Immunomodulatation of the elderly immune system

The progressive age-related dysregulation and decline of the immune system is known as “immunosenescence”. In the aging immune system, there is an accumulation of memory cells as a result of chronic stimulation with repeated clinical and subclinical infections [13]. This chronic stimulation causes these memory cells to become exhausted and no longer able to function, contributing to an increased susceptibility of elderly to infectious pathogens [79, 80]. The aging immune system is also characterized by a decline in the numbers of naive T cells, an imbalance in Th1/Th2 cell subsets, and a decrease in T cell receptor (TCR) repertoire [75, 81, 82].

A cellular immune response with release of Th1 (type 1) cytokine profile, including IFN-ϒ, TNF-α and IL-2, is known to be protective against most viral infections [83]. IFN-ϒ in particular has been shown to play an essential role in clearance of viral infection [84]. Modulating the immune system of elderly individuals through alloantigen priming to provide high titers of non-exhausted Th1/CTL memory cells that can be non-specifically activated upon encounter with any virus to cause release type 1 cytokines may provide an immediate anti-viral immune response upon viral exposure and could also reinstate responsiveness to viral vaccines [85]. The delay in IFN responses to virus infection in the elderly is due to active suppression by viral proteins and immunosenescence [86], which allows virus to reach high titers early in infection, leading to immunopathology, T-cell apoptosis and progression to lethal pneumonia [87, 88].

The immediate release of IFN-ϒ upon acute viral infection corrects the problem of delayed immune responsiveness and creates an early “anti-viral state” [89, 90]. Establishment of the anti-viral state with early release of IFN-ϒ provides a crucial initial line of defense against viral infection [91]. The anti-viral state is normally induced through early release of type I/III IFN from viral infected cells [92]. However, many viruses, including coronavirus, can suppress production of type I/III IFN as an immune avoidance mechanism [93–96]. The strategy of providing a pool of non-exhausted memory cells which produce Type II IFN (IFN-ϒ) upon viral encounter, overcomes the viral escape. IFN-ϒ creates an anti-viral state even in type I/III IFN resistant cells [97]. Further, IFN-ϒ mobilizes innate effector cells that can respond rapidly to eliminate viral infection [98] and also produce IFN-ϒ. Thus, early IFN-ϒ response to viral infection through allo-priming provides a rapid means of control over viral infection in the elderly.

Chronic inflammation in the elderly is known to be associated with increased susceptibility to cancer [99, 100], atherosclerosis [101] and neurodegenerative disorders [102, 103]. The modulation of the elderly immune system that occurs by allo-priming can also have the effect of counter-regulating resident chronic inflammation [104, 105] which causes “inflammaging” resulting in reversing of immunosenescence [106]. Thus, allo-priming protocols may not only provide universal anti-viral protection for the elderly, but may also serve as an “anti-aging” mechanism to protect from co-morbid diseases of aging.

### Clinical features of viral infection in elderly

Allo-priming is predicted to reduce the severity of clinical symptoms upon encounter with virus. Clinical features of respiratory viral infections in humans vary from a first subset that experience mild flu-like symptoms which subside over a few days; to a second subset that experience moderate to severe flu-like symptoms associated with high fever, hypoxemia and progression to pneumonia-like symptoms. These symptomatic patients are at high risk to progress to acute respiratory distress syndrome (ARDS), sepsis, multiple organ failure and death. The subset with mild flu-like symptoms is associated with younger healthy individuals, while the subset associated with more severe symptoms is mostly associated with elderly adults that are frail and/or present with co-morbidities.

The progression to more serious symptoms can occur even when there is a decline in virus titers [107] connected to a late over-exuberant immune response. The initial delayed immune response leads to high viral titers which is followed by an exaggerated late immune response and a “cytokine storm”. This sequence of events is believed to be responsible for the severe symptoms related to respiratory virus infections in the elderly.

During pathogenic virus infection, a cytokine storm leads to excessive inflammatory infiltrates and virus-induced tissue destruction which contributes to morbidity and mortality. The timing and source of the IFN in the cytokine storm can affect the clinical course of viral disease. High serum levels of IFN-ϒ late in the course of viral infection is correlated with more severe disease [108] and immunopathology [109], whereas early IFN release results in lower viral titers and less severe disease. These observations support the prediction that early activation and release of IFN-ϒ from non-exhausted allo-specific memory Th1/CTL will correct this problem and avoid progression to severe disease.

The type of cytokine storm associated with severe disease is caused by cytokines released by activated monocytes, producing a storm of IFN-α, CCL-2, IL-6, TNF-α, CCL-3, CCL-5, CxCL-2, IL-1α, and IFN-γ. This monokine cytokine storm is directly correlated with morbidity and mortality [110]. The majority of fatalities associated with cytokine storm also developed bacterial pneumonia and sepsis. It is recognized that a major cause of respiratory failure is coexistent bacterial pneumonia leading to ARDS. ARDS is characterized by damage to the endothelial–epithelial barrier of the alveoli, resulting in fluid leakage and accumulation in the alveolar lumen inhibiting gas exchange.

The elderly have a pre-disposition to develop progressive respiratory compromise and sepsis. In fact, sepsis is considered a disease of aging [111] and is among the top causes of ICU admissions of the elderly. The features of sepsis-induced immunosuppression, independent of age, share many of the same characteristics of immunosenescence. During the last decades, there has been a significant increase in incidence of sepsis in patients over 65 years of age [112]. Many respiratory virus patients that progress to develop pneumonia and ARDS [113, 114] later die due to sepsis.

The overactive immune response to sepsis, including monokine “cytokine storm” results from over activated M1 macrophages in response to tissue damage [115]. The response to sepsis is similar between old and younger patients. However, the mortality is much higher in older patients [116]. The difference is related to the dysregulated immune systems of the elderly. The elderly are unable to turn down the over-activated monocyte response, leading to disease progression, while regulatory mechanisms in the young counter-act and prevent the consequences of monocyte over-activation.

The dysregulated immune system of the elderly is related to the increased activity of myeloid-derived suppressor cells (MDSC) [117, 118]. MDSCs are potent inducers of immunosenescence [119], sepsis-acquired immunodeficiency [120] as well as cancer metastasis and progression [121]. MDSC are positively correlated with IL-6 and negatively correlated with IFN-ϒ [122, 123].

Immunosenescence causes an imbalanced homeostatic regulation of innate and adaptive immune responses, diminishing the host capacity to rapidly restore balanced immune functions. Thus, combined effects of age-induced immunosuppression, delayed innate immune responses [86], exaggerated late immune responses due to altered homeostasis [88], all combine to make the post-viral infection period in the elderly have a longer duration. As a result, there are increased levels of viral propagation, higher incidences of tissue injury, and increased progression to ARDS and septic shock in the elderly.

Accordingly, allo-priming enabling the early release of IFN-ϒ in response to viral infection can prevent the differentiation of MDSC and counter-regulate resident MDSC immunosuppression in the elderly [124]. This will have the effect of remodeling the elderly immune system in a manner which will prevent the severe symptoms of viral infection and progression to ARDS and sepsis.

## Conclusion

Highly pathogenic human CoVs pose a substantial threat to public health. There are basically two groups of patients upon exposure to CoV, with the majority developing a short duration of clinical symptoms and the minority experiencing severe disease characterized by pneumonia and ARDS. The elderly are disproportionately vulnerable to severe disease due to immunosenescence and comorbidities. It is likely that CoVs will continue to cross species and cause additional outbreaks in the future. Therefore, even if a vaccine is developed for prevention of COVID-19, it would not protect against the next outbreak of a novel virus. Therefore, there is a need to develop novel strategies, not only to control the current pandemic, but also to be prepared to prevent the next pandemic.

Strategies to develop a vaccine to elicit neutralizing antibodies to SARS-CoV-2 are technically challenging due to the conformational hiding of the receptor binding domain (RBD) and the ability of this virus to transfer cell-to-cell in syncytia and infect target cells in a ACE2- and protease-independent manner by pinocytosis, limiting environmental exposure needed for antibody neutralization activity. Development of an anti-viral vaccine against complex viruses, such a SARS-Cov-2, is likely to require a potent and broad T-cell response to overcome viral escape mechanisms related to humoral immunity.

A vaccine that elicits a robust cellular immune response requires identification of conserved viral epitopes and effective processing and presentation of viral antigens by APC on MHCI and MHCII in conjunction with co-stimulatory signals, as well as a diversity in the naive T cell repertoire. In addition, CTL memory cells resulting from vaccination will require infected cells to present the selected viral antigens in the vaccine on MHCI. Selection of common viral epitopes which present on MHC I (and MHC II) for vaccine development is technically difficult and CoV infection causes the down-regulation of MHC I on infected cells, making infected cells invisible to CTL. Since the elderly lack naïve T-cells that are necessary for development of CTL with broad specificity to viral antigens, elderly would be less likely to respond efficiently to any future vaccine which targets cellular immunity.

The rejection of allografts is one of the most conserved and powerful immune mechanisms, making priming with alloantigens an ideal candidate for use in vaccination of the elderly. AlloStim^®^ is in use under a US FDA cleared IND and has been shown to readily be rejected by heavily pre-treated, immunosuppressed metastatic cancer patients. A phase I/II clinical trial protocol for use of AlloStim^®^ in healthy elderly adults in currently under review by US FDA. Upon clearance, clinical trials could be initiated to evaluate this allo-priming concept in short order. For proof-of-concept, longitudinal PBMC samples are proposed to be collected pre- and post-allo-priming. The PBMC are to be pulsed with a panel of inactivated viruses or recombinant viral proteins to determine if they will non-specifically activate the allo-specific Th1/CTL memory cells. The supernatants from the viral-pulsed PBMC are proposed to be evaluated in live virus cytolytic plaque assays to determine if viral propagation is suppressed.

Elderly persons are particularly susceptible to progressive viral disease and have delayed immune responses to viral infection due, at least in part, to immunosenescence, Th2 immune bias, decreased diversity of naïve T-cells and high frequencies of exhausted memory cells from chronic inflammation (e.g., CMV). This results in a natural loss of the ability to mount effective innate and adaptive cellular immune responses to invading pathogens. The delayed and suppressed cellular immune response in the elderly enables viruses to become widely established and the resulting increased viral titers causes dys-regulated immune responses that can lead to a late, over-active immune response which can progress to pneumonitis, multiple organ failure and death.

The key to an effective natural innate immune response that can limit the course of virus infection is the early production of type I/III interferons (IFN) and subsequent switch to type II IFN (IFN-ϒ) production as the immune response matures. Successful viral infections become established due, in large part, to delayed innate immune responses and viral-mediated suppression of type I/III IFN production, allowing for rapid viral propagation which eventually results in dys-regulated immune responses which are correlated with tissue destruction, co-infection, multiple organ failure and death.

To prevent accumulation of high viral burden in the elderly upon viral infection, allo-priming provides a mechanism whereby a ready pool of de-novo primed T-cells are in circulation that can respond rapidly to viral infection by producing IFN-ϒ. The presence of non-exhausted Th1/CTL memory immune cells will modify the elderly immune character by providing a Th1 re-balancing mechanism in the memory cell compartment. This is accomplished through the creation of a high titer of polyclonal, allo-specific, non-exhausted, memory Th1/CTL T-cells through intradermal injections of activated allogeneic Th1-like cells (AlloStim^®^). The allo-specific memory cells resulting from the priming are programmed to produce IFN-ϒ upon activation. IFN-ϒ has a direct anti-viral effect on cells infected with virus and can also protect uninfected cells from infection. IFN-ϒ creates the same anti-viral environment as innate release of Type I/III IFN does in an effective natural innate immune response.

These allo-specific memory cells are capable of cross-reacting with foreign viral antigens and can be readily activated non-specifically by environmental stimuli such as cytokines and foreign pathogens. The memory pool of allo-specific immune cells in primed individuals can also be re-activated by additional intradermal or intravenous infusion of AlloStim^®^ allogeneic cells upon first symptoms of viral disease. The by-stander effect of allo-specific Th1/CTL memory T-cell activation and IFN-ϒ production is predicted to elicit protective effects on cells in the respiratory tract and generate rapid immune-mediated viral clearance as well as condition the microenvironment in infected tissues (e.g., lung epithelial cells) for an in situ vaccination leading to viral-specific immunity and memory that is specific for the invading virus. The heterologous immune effect can amplify the pan-viral protection upon each viral encounter, providing the possibility of long-term protection.

The proposed alloantigen priming strategy can also be used in conjunction with any viral-specific vaccines. Co-injection of allo-specific and viral specific antigens could accelerate the adaptive cellular immune response to a known virus and serve as a means to enhance response to vaccines in the elderly. Subsequent injection of allogeneic cells will stimulate an allo-rejection memory recall response which can non-specifically activate resident viral-specific cells elicited by vaccination. This dual activation provides a mechanism to upregulate MHCI on infected targets, making the memory cells elicited by viral-specific vaccination able to identify and kill viral infected targets. The combination approach also has the advantage of overcoming the narrow immunity conferred by a single peptide vaccine by incorporating the in situ vaccination mechanism to elicit broad viral-specific cellular immune responses.

This allo-priming mechanism can also be used as a counter-measure to bio-terrorism and bio-economic terrorism. Individuals that have been primed with alloantigen could be treated with an emergency injection of alloantigen to activate innate immunity and initiate the cascade of immune events leading to clearance of an unknown pathogen. Alloantigen could be provided in syringes for emergency ID injection upon concern for bioweapon exposure or first occurrence of symptoms, much in the same manner as epinephrine is provided to prevent anaphylactic shock.

While finding methods to treat and prevent COVID19 infection is of urgent priority, it is also important to consider that the current pandemic is the third major outbreak of a novel coronaviral infection in humans within the past 20 years. Currently there are no registered vaccines or means of therapeutic protection against the prior SARS or MERS outbreaks available anywhere in the world. This is despite considerable efforts from experts worldwide to develop vaccines. The same technical obstacles preventing development of specific vaccines and treatments for these past CoV outbreaks likely also exist for development of a vaccine for the current COVID-19 viral pandemic. However, even if an effective vaccine is developed, it is estimated that it will take at least a year to complete clinical testing sufficient to obtain regulatory approval. If COVID-19 fades as did SARS and MERS, by the time a vaccine is available it will no longer be as urgent.

However, protection of the vulnerable elderly population from the next pandemic and protecting the world economy from natural and bioweapon threats will remain an urgent need. Therefore, novel approaches to addressing the problem of new emerging viral epidemics, especially in the vulnerable elderly population, are of a high priority. Allo-priming holds promise as such an approach.

## Data Availability

Not applicable.

## Abbreviations

ARDS: Acute respiratory distress syndrome
CMV: Cytomegalovirus
CoV: Coronavirus
CTL: Cytotoxic T-lymphocyte
CCL-: C–C motif ligand
CxCL-: CxC motif ligand
DAMP: Danger associated molecular pattern
DC: Dendritic cell
FDA: Food and Drug Administration
GM-CSF: Granulocyte-macrophage colony stimulating factor
GRP: Glucose-regulated protein
GVHD: Graft vs. host disease
GVT: Graft vs. tumor
HIV: Human immunodeficiency virus
HCV: Hepatitis C virus
HSP: Heat shock protein
ID: Intradermal
IFN: Interferon
IL: Interleukin
IND: Investigational New Drug
MDSC: Myeloid-derived suppressor cell
MERS: Middle East Respiratory Syndrome
MHC: Major histocompatibility complex
NK: Natural killer cell
NKT: Natural killer T cell
US: United States
SARS: Severe acute respiratory syndrome
Th1: T-helper 1
TNF: Tumor necrosis factor
